# HIV-1 and alcohol abuse promote astrocyte inflammation: A mechanistic synergy via the cytosolic phospholipase A2 pathway

**DOI:** 10.1038/cddis.2015.346

**Published:** 2015-12-10

**Authors:** R Pandey, A Ghorpade

**Affiliations:** 1 Department of Cell Biology and Immunology, University of North Texas Health Science Center, Fort Worth, TX 76107, USA

Over the past 35 years, the HIV/AIDS pandemic cost our society about 30 million lives, and yet another 34 million more continue to live with the disease. Despite significant efforts, both the incidence and the prevalence of HIV-1 disease are on the rise (http://www.unaids.org/en/resources/campaigns/20121120_globalreport2012). Nearly 2.5 million new cases are reported each year globally, the same number of lives that succumb to alcohol abuse annually (http://www.who.int/substance_abuse/publications/global_alcohol_report/msbgsruprofiles.pdf). The World Health Organization, lists alcohol consumption as the third greatest risk factor for disease and disability worldwide, including increased risk of HIV-1 transmission.^1^ The term HIV-associated neurocognitive disorders (HAND) characterizes a group of ailments of varying degrees of impairment of cognition and associated functioning in HIV-infected individuals.

To the general public on main street, alcohol consumption is a recreational activity that produces pleasurable feelings such as euphoria, reward and a sense of well-being attributed to serotonin, dopamine and other neurotransmitter release. The National Institute on Alcohol Abuse and Alcoholism (NIAAA) has set drinking parameters that are often ignored, leading to binge or heavy drinking. In many instances, this culminates in alcohol-use, -abuse or -dependence disorder, commonly referred to as ‘alcoholism', which is often seen in HIV-1-infected patients (http://www.niaaa.nih.gov/news-events/news-releases). Our recent study attempts to shed new mechanistic light on the cellular and molecular processes involved in the combined effects of HIV-1 and alcohol on astrocytes, the numerically superior cells in the brain and the first responders to any CNS insult.^2^

Significantly impaired memory and executive functions are reported in both HIV infection and chronic alcoholism.^3^ Our study builds on the existing literature about individual effects of alcohol (EtOH) or HIV-1, and uncovers a novel signaling pathway centered on cytosolic phospholipase A_2_ (cPLA_2_), in the combined setting.^2^ At the cellular level, in addition to direct effects on neuronal cells, alcohol activates NF-*κ*B signaling and other inflammatory pathways to induce cytokines and inducible nitric oxide synthase in mouse astrocytes.^4^ *In vivo*, alcohol induces TLR4- and IL-1R-mediated activation of multiple signaling pathways leading to inflammation.^4^ Prenatal and postnatal ethanol exposure is known to elevate oxidative stress either through free radicals (ROS/RNS) or disruption of antioxidant mechanisms, thereby promoting apoptosis.^5^ Mitochondrial oxidative damage releases cytochrome *c* and activates caspases, ultimately causing cell death. Alcohol exposure also results in increased cytochrome P450-2E1 (CYP2E1) activity, ROS production and secretion of prostaglandin E2 (PGE2) in primary human astrocytes.^6^

Scientific literature is strewn with a plethora of studies, reports and investigations regarding the importance of inflammation as a key common factor in multiple diseases. Even the popular magazine ‘TIME' has discussed and debated inflammation, and how it links to a number of diverse diseases such as Alzheimer's disease, heart attack and cancer.^7^ Non-steroidal anti-inflammatory drugs including, but not limited to, aspirin, ibuprofen and the notorious cyclooxygenase 2 (COX2) inhibitors: rofecoxib, valdecoxib and celecoxib have been used to treat a wide variety of diseases. Historically, COX2 inhibitors have been popular, yet controversial therapeutic options. It is well established that secretion of PGE2 parallels increase of PLA_2_ and COX2.^6^ Our study uncovers that cPLA_2_ signaling, directly upstream of COX2, is activated in astrocytes exposed to alcohol and/or HIV-1. Furthermore, cPLA_2_ activation in human astrocytes led to greater levels of arachidonic acid (AA), a common pro-inflammatory and neurotoxic mediator.^2^

With the widespread use of anti-retroviral therapy (ART), the incidence of cognitive and motor deficits related to HIV-1 infection (HIV-associated dementia) has declined, nevertheless, neuropsychological deficits continue.^8^ HIV-1 infection frequently results in cognitive impairments, collectively known as HAND. NIAAA raised serious concerns regarding increased cases of alcohol abusers and HIV-1 infections, revealing alcohol may interact with ART medications and/or exacerbate adverse effects of these medications. Several reports demonstrate that alcohol, HIV-1 and other HAND-relevant stimuli generate excessive pro-inflammatory molecules through a number of signaling pathways resulting in neuroinflammation.^9, 10^ In addition, the number of drinks consumed have a direct relationship with higher relative risk for stroke in a human cohort. A few prior reports suggest that alcohol exacerbates inflammatory responses in the setting of HIV-1,^6^ whereas multiple investigations demonstrate independent effects of astrocyte activation with EtOH, HIV-1 or HAND-relevant stimuli such as IL-1*β* and TNF-*α*.^9, 11^

Despite the knowledge that alcohol exacerbates apoptosis, oxidative stress and inflammatory episodes, key mechanistic questions remain unanswered regarding combined effects of EtOH and HIV-1. In particular, (1) what are the effects of acute *versus* chronic alcohol on CNS HIV-1 disease progression? (2) how does alcohol induce inflammation in human astrocytes in context of HIV-1?; and last (3) how can we capitalize on the insight into these mechanisms towards development of therapeutic options to address both problems?

To these ends, our recent article reports that alcohol and HIV-1 co-treatment not only decreases cell viability, proliferation and increases apoptosis, but also leads to mitochondrial depolarization.^2^ Mitochondria play a critical role in cell injury by regulating power stores and contributing to oxidative stress and cell death during disease. TNF-*α*-associated cytotoxicity involves the early loss of mitochondrial function, as demonstrated by mitochondrial depolarization due to permeability transition pore opening in hepatocytes from alcohol-fed animals.^12^ In addition, AA-induced toxicity in CYP2E1-overexpressing hepatocytes is associated with the convergence of lipid peroxidation, intracellular Ca^2+^ release and activation of cPLA_2_.^12^ Using primary human astrocytes as *in vitro* models, we show increased mitochondrial depolarization and apoptosis accompanied by concomitant induction of cPLA_2_/CYP2E1 and enhanced AA release. Taken together, these mechanisms can directly contribute to oxidative stress and mitochondrial dysfunction in astrocytes during HAND-associated alcohol abuse.^2^

Our study not only provides detailed analyses regarding the role of alcohol in the setting of HIV-1, but more importantly, we shed new light on mechanisms through which alcohol and HIV-1 synergize to exacerbate detrimental processes leading to neurodegeneration. Our work underpins the concept that alcohol in HIV/AIDS has a critical role in regulating inflammation, mitochondrial dysfunction and cytotoxicity that can culminate in neurodegenerative outcomes. Furthermore, we have highlighted key unanswered questions and identified several new lines of investigations that will provide vital clues to answer them. Figure 1 illustrates the intricate signaling cascades where cPLA_2_ serves as a focal point through which EtOH and/or HIV-1 increase AA, the downstream target of cPLA_2._ Likewise, COX2 increased upon exposure of EtOH and HIV-1 alone or in combination. Interestingly, our data suggest that targeting cPLA_2_ upstream of AA and COX2 will likely have greater impact in controlling inflammatory neurological outcomes in the context of HAND and alcohol abuse co-morbidity.^2^

These results will provide a better insight into how alcohol abuse affects neuroinflammation in the individuals living with HIV-1 and also will aid in the development of new therapeutic strategies to prevent alcohol-mediated effects in HIV-1-infected population. Although it is enticing to suggest that these approaches will likely have broader implications in neuroinflammation in general, our investigation primarily addressed alcohol effects limited to the context of HAND. Our future works will focus on elucidating cPLA_2_ upstream intracellular pathways. In addition, we will investigate the functional outcomes, such as glutamate imbalance or other neurotransmitter release, and how they are associated with excitotoxicity, leading to neuronal dysfunction. We expect that our story will prove exciting in future research endeavors to design therapeutic interventions targeting astrocytes to successfully mitigate neuroinflammation, and thereby protect neurons during alcohol abuse and HAND.

## Figures and Tables

**Figure 1:**
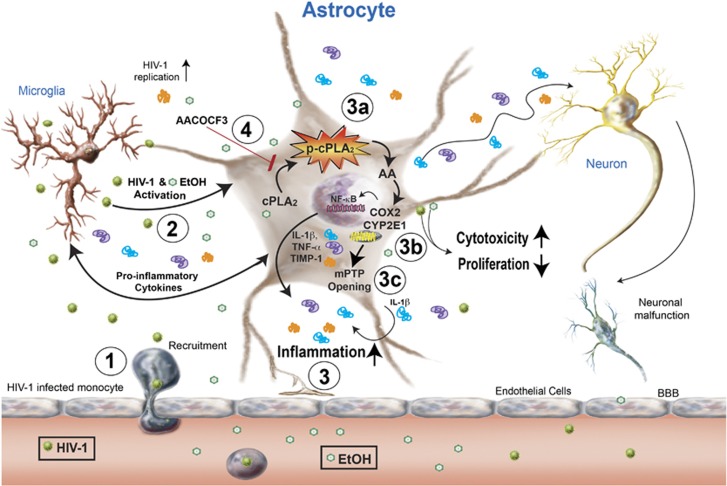
Alcohol and HIV-related neuroinflammation and cytotoxicity contribute astrocyte cPLA_2_ activation into the limelight. Alcohol (EtOH), fat- and water-soluble dangerous teratogen, crosses the blood brain barrier, whereas HIV-1 invades the brain via infected monocytes. (1) Infected microglia (resident macrophages of the brain) and local HIV-1 reservoirs are activated and secrete cytokines, virus and other neurotoxic agents, such as viral proteins and ROS/or reactive nitrogen species. (2) Astrocytes, in turn, are activated perpetuating neuroinflammation leading to neurodegeneration. (3) Our data established that how alcohol and HIV-1 activate astrocytes regulate inflammation. EtOH and HIV-1 alone or in combination induce cPLA_2_ phosphorylation, thereby inducing hydrolytic release of AA from membrane phospholipids. (3a) AA is metabolized by COX2 and CYP2E1 enzymes into eicosanoids, such as prostaglandin E2 (PGE2), leading to neuroinflammation via *NF*-*κ*B. (3b) In addition, EtOH and/or HIV-1 increased mitochondrial damage, analyzed as mPTP opening. (3c) Arachidonyl-tri-flouro methyl ketone (AACOCF3), a cPLA_2_ specific inhibitor, blocked this signaling process thereby diminishing inflammatory responses. (4) We propose that cPLA_2_ signaling is a critical regulator in alcohol and HAND co-morbidity and a promising future therapeutic target
